# Inter-Dependent Mechanisms Behind Cognitive Dysfunction, Vascular Biology and Alzheimer's Dementia in Down Syndrome: Multi-Faceted Roles of APP

**DOI:** 10.3389/fnbeh.2015.00299

**Published:** 2015-12-01

**Authors:** Dean Nizetic, Christopher L. Chen, Wanjin Hong, Edward H. Koo

**Affiliations:** ^1^Lee Kong Chian School of Medicine, Nanyang Technological University, SingaporeSingapore; ^2^The LonDownS Consortium, Wellcome TrustLondon, UK; ^3^The Blizard Institute, Barts and The London School of Medicine, Queen Mary University of LondonLondon, UK; ^4^Department of Psychological Medicine and Memory Aging and Cognition Centre, National University Health System, SingaporeSingapore; ^5^Department of Pharmacology, National University of Singapore, SingaporeSingapore; ^6^Agency for Science, Technology and Research (A^*^Star), Institute of Molecular Cell Biology, SingaporeSingapore; ^7^Departments of Medicine and Physiology, Yong Loo Lin School of Medicine, National University of Singapore, SingaporeSingapore; ^8^Department of Neurosciences, University of California, San DiegoSan Diego, CA, USA

**Keywords:** Down syndrome, vascular dementia, neuron activity-dependent, cognitive dysfunction, amyloid beta-peptides, amyloid beta-protein precursor

## Abstract

People with Down syndrome (DS) virtually all develop intellectual disability (ID) of varying degree of severity, and also have a high risk of early Alzheimer's disease (AD). ID prior to the onset of dementia, and its relationship to the onset of dementia in DS is a complex phenomenon influenced by many factors, and scarcely understood. Unraveling the causative factors and modulators of these processes remains a challenge, with potential to be informative for both ID and AD, for the development of early biomarkers and/or therapeutic approaches. We review the potential relative and inter-connected roles of the chromosome 21 gene for amyloid precursor protein (APP), in both pathological conditions. Rare non-DS people with duplication of APP (dupAPP) get familial early onset AD (FEOAD) with virtually 100% penetrance and prominent cerebrovascular pathology, but don't suffer from ID before dementia onset. All of these features appear to be radically different in DS. On the other hand, rare individuals with partial trisomy 21 (T21) (with APP, but not DS-critical region in trisomy) have been described having ID. Likewise, partial T21 DS (without APP trisomy) show a range of ID, but no AD pathology. We review the multi-faceted roles of APP that might affect cognitive functioning. Given the fact that both Aβ secretion and synaptic maturation/plasticity are dependent on neuronal activity, we explore how this conflicting inter-dependency might affect cognitive pathogenesis in a dynamic way in DS, throughout the lifespan of an individual.

## Introduction

Virtually all people with Down syndrome (DS) show some degree of intellectual disability (ID), due to many factors, including a certain degree of cognitive dysfunction caused by the pathobiology of trisomy 21 (T21) (Epstein, 2002; Head et al., 2012). On the other hand, T21 is the most common known genetic cause of obligatory development of pathological hallmarks of AD in the brain tissue (Mann, 1988a; Lott and Head, 2005). This happens extremely early in virtually all people with DS (in the 30-s) (Mann, 1988a,b). The cause of this is apparently an extra copy of APP (amyloid precursor protein gene located on chromosome 21), as one individual with DS at age 74 (with no dementia, and no amyloid pathology in the brain) has been described who was born with partial chromosome 21 trisomy that did not include APP (Prasher et al., 1998). In rare families of non-DS (euploid) people, APP micro-duplications (dupAPP) are responsible for Familial Early Onset Alzheimer's Disease (FEOAD), with virtually 100% penetrance by age 60 and a median onset age of clinical dementia of 41–51, and dupAPP has so far never been seen in any human unlinked to FEOAD (Rovelet-Lecrux et al., 2006, 2007; Sleegers et al., 2006; Kasuga et al., 2009; Thonberg et al., 2011; Cohn-Hokke et al., 2012; McNaughton et al., 2012; Wallon et al., 2012). In adults with DS, the average age at onset of dementia varies greatly, with ~25% starting extremely early (in early thirties), but a good 25–50% having a much delayed onset (compared to dupAPP), or not developing dementia at all by age >60 (Holland et al., 1998; Sekijima et al., 1998; Tyrrell et al., 2001; Coppus et al., 2006; McCarron et al., 2014). Also, cerebrovascular pathology, mainly intra-cerebral hemorrhage is a prominent symptom of dupAPP (McCarron et al., 1998; Rovelet-Lecrux et al., 2006; Sleegers et al., 2006; Kasuga et al., 2009; McNaughton et al., 2012; Wallon et al., 2012), whereas it is rarely seen in DS (Belza and Urich, 1986). This is in spite of abundant deposits of amyloid in blood vessels (congophilic angiopathy) in both conditions (see below). On the other hand, people with dupAPP largely have a normal development, intellectual and social functioning (families) prior to dementia, in contrast to people with DS.

This indicates that mechanisms are in action in DS individuals (that dupAPP individuals don't have), that cause ID, can accelerate clinical onset of dementia, as well as factors that can delay onset or protect from overt dementia and associated intra-cerebral hemorrhage.

While dupAPP patients, by definition, are seen in families exhibiting FEOAD, this implies that this is a self-selected group of people that largely lack ID prior to dementia. On the other hand, rare individuals with partial T21 (with APP, but not DS-critical chr21 region in trisomy) have been described having I.Q. ranging from ~30 to <80, prior to dementia-prone age (Korbel et al., 2009; summarized in Table 1). This could be caused by an overdosed action of APP, but equally other genes included in the partial trisomy, mostly located in the “gene poor” proximal half of chromosome 21 (Groet et al., 1998). We review potential roles for APP, that go beyond the AD paradigm, and could contribute to the modulation of ID.

**Table 1:** **Effects of the trisomy of APP and other segments of human chromosome 21 on development of intellectual disability (independently of dementia), and on presence of early clinical dementia**.

**Human genotype/ Mouse model**	**T R I S O M Y OF:**			**Intellectual disability independent of dementia**	**Early Clinical dementia < age55**	**References**
	**APP**	**“DS-critical Region”**	**Other parts of HSA21**			
Down syndrome (DS)	Yes	Yes	Yes	Yes	~60%	Summarized in Wiseman et al., 2015
DS-partial trisomy	Yes	Yes	Yes	Yes	?	Summarized in Korbel et al., 2009
DS-partial trisomy	No	Yes	Yes	Yes	No	Prasher et al., 1998
Non-DS-partial trisomy	Yes	No	Yes (large)	Yes	?	Park et al., 1987; Korbel et al., 2009
Dup-APP (majority)	Yes	No	Yes (limited)	No	>99%	Rovelet-Lecrux et al., 2006, 2007; Kasuga et al., 2009; Thonberg et al., 2011; Cohn-Hokke et al., 2012; McNaughton et al., 2012; Wallon et al., 2012
Dup-APP-only	Yes	No	No	No	>99%	Sleegers et al., 2006
Ts65Dn	Yes	Yes	Yes	Yes^*^	Not applicable	Reeves et al., 1995
Ts1Rhr	No	Yes	No	Yes^*^	Not applicable	Belichenko et al., 2009
Tc1	No	Yes	Yes	Yes^*^	Not applicable	O'Doherty et al., 2005; Morice et al., 2008

“*?”, published data are missing. “*

* *”, mouse phenotypes equivalent of human ID, such as learning, memory, electrophysiological, and behavioral defects. Mouse models of trisomy 21 alone do not reproduce Alzheimers pathology in the brain, or signs of progressive neurodegenerative phenotypes (therefore “Not applicable” entry)*.

## Beyond Alzheimer's: multiple roles of APP potentially affecting cognitive dysfunction in DS (summarized in Table 2)

T21 causes synaptic plasticity defects and dendritic spine abnormalities in human brains (Marin-Padilla, 1976; Ferrer and Gullotta, 1990) as early as 19 weeks *in utero* (Weitzdoerfer et al., 2001). This pathology can be modeled using mouse models of DS (Haas et al., 2013). Dendritic spines and their plasticity are also the site of important pathology in neurodevelopmental conditions such as Rett and FraX syndromes (Troca-Marín et al., 2012; Chang et al., 2013). Their loss contributes to pathogenesis of AD and PD (McGowan et al., 2006; Schulz-Schaeffer, 2010), though specific morphological differences and causes are likely unique to each condition. Generation and deposition of beta-amyloid peptides (Aβ40 and Aβ42) is linked with destruction of dendritic spines and synaptic loss in AD (McGowan et al., 2006; Shrestha et al., 2006). APP protein (Kamenetz et al., 2003), as well as other chromosome-21 encoded gene products, may have important functions in synaptic biology (Wang et al., 2013).

**Table 2:** **An overview of a variety of processes affected by an increased dose of APP protein and/or its derivative Aβ peptides, that may contribute in DS to pathogenesis of Alzheimer's dementia or cognitive impairment (I.D.), or both**.

**Process affected**	**APP**	**Aβ peptides**	**References**
Dendritic spines destruction	No	Yes	McGowan et al., 2006; Shrestha et al., 2006
Synapse loss	No	Yes	Kamenetz et al., 2003; McGowan et al., 2006; Scheff et al., 2006; Shrestha et al., 2006
GABA-ergic short-term plasticity dysfunction	Yes	No	Yang et al., 2009
Astrocytic glutamate release	Yes	Yes	Talantova et al., 2013
Extra-synaptic NMDA receptor activation	Yes	Yes	Innocent et al., 2012; Talantova et al., 2013
Receptor-mediated synaptotoxicity	No	Yes	Benilova and De Strooper, 2013
Its levels are increased by synaptic activity	No	Yes	Cirrito et al., 2005; Sullivan et al., 2013; Cheng et al., 2014
Adult hippocampal neurogenesis	Yes	No	Wang et al., 2014b
Retrograde neurotrophin signaling	Yes	No	Salehi et al., 2006
Cholinergic forebrain neuronal degeneration	Yes	No	Salehi et al., 2006
Increased levels and re-distribution of phosphorylated Tau	Yes	Yes	Israel et al., 2012; Shi et al., 2012; Moore et al., 2015
Cerebral amyloid angiopathy	Yes	Yes	Belza and Urich, 1986; McCarron et al., 1998; Rovelet-Lecrux et al., 2006, 2007; Kasuga et al., 2009; Cohn-Hokke et al., 2012; McNaughton et al., 2012; Wallon et al., 2012; Nicolas et al., 2015
Intra-cerebral hemorrhage	Yes	No	McCarron et al., 1998; Rovelet-Lecrux et al., 2006, 2007; Kasuga et al., 2009; Cohn-Hokke et al., 2012; McNaughton et al., 2012; Wallon et al., 2012

*List of references is not exhaustive for all processes, publications best illustrating the point were selected*.

Down's syndrome causes overexpression of miR-155, a chromosome 21–encoded microRNA that negatively regulates C/EBPb, thereby reducing sorting nexin 27 (SNX27) expression and resulting in synaptic dysfunction (Wang et al., 2013). SNX27 is a novel activity-dependent signaling molecule that has the ability to decode the Ras signal and transduce the plasticity stimuli to the delivery of postsynaptic AMPA receptors (Loo et al., 2014). So, SNX27 signaling is also activity-dependent: the more neuronal activity, the bigger chances of seeing pathology due to inability to raise sufficient SNX27 levels. On the other hand, SNX27 acts as a γ-secretase interaction partner to promote dissociation of the γ-secretase complex, thus decreasing its proteolytic activity, thereby reducing the generation of all Aβ peptides (Wang et al., 2014a). The effects of the apparent reduction of SNX27 in DS would therefore be expected to further increase Aβ levels, (by lessening the dissociation of the γ-secretase complex), and therefore worsen AD-pathology. However, the end results of this on AD pathogenesis are far from simple, and need to be carefully further investigated. While inhibition of γ-secretase activity might reduce the levels of neurotoxic Aβ42, intriguingly, very recent results on hiPSC-derived neurons show that chemical inhibition of γ-secretase activity in T21 neurons dramatically increased levels of Tau, the principal constituent of neurofibrillary tangles. This could actually have pro-dementia effects (Moore et al., 2015). The picture is even more intriguing, as the same study found that addition of a γ-secretase modulator (GSM), E2012, decreased Tau levels in T21 neurons (that showed an otherwise increased Tau levels; Moore et al., 2015).

Synaptic dysfunction is an early feature of AD, likely much before significant Aβ deposition (Arendt, 2009). There is a line of thought that AD neurodegenerative processes begin by alterations in synapse function/structure leading to synapse loss, prior to significant neuronal loss. Consistent with this, disturbance in synaptic integrity is detected in patients with mild cognitive impairment (MCI), which is sometimes perceived as an early stage of AD (Scheff et al., 2006). Loss of synaptic markers is a predictor of disease progression in AD (Selkoe, 2002). Aβ, generated from proteolytic processing of APP, has been shown to disrupt synapses (Kamenetz et al., 2003; Scheff et al., 2006; Shrestha et al., 2006) and, conversely, synaptic activity is an important factor regulating Aβ levels (Cirrito et al., 2005; Sullivan et al., 2013; Cheng et al., 2014). Aβ was also found to induce astrocytic glutamate release, extra-synaptic NMDA receptor activation, and synaptic loss (Talantova et al., 2013).

APP and Aβ have other, direct and indirect roles in functioning of synapses. Overexpression of APP affects Ca_*v*_1.2 L-type calcium channel levels and through this influence GABAergic short-term plasticity (Yang et al., 2009). APP may contribute to postsynaptic mechanisms via the regulation of the surface trafficking of excitatory N-methyl-D-aspartate (NMDA) receptors (Innocent et al., 2012). Aβ was also shown binding to a number of receptors embedded in neuronal plasma membrane, (such as PrP, EphB2, FcγRIIb, and PirB), potentially contributing to receptor-mediated synaptotoxic pathways (Benilova and De Strooper, 2013).

There are also compelling evidence for alterations in synaptic function/plasticity in DS mouse models. For instance, altered excitatory/inhibitory balance is known to modify synaptic plasticity in DS mouse models (Kleschevnikov et al., 2004; Souchet et al., 2014). Importantly, increased APP expression in mouse models of DS has other deleterious effects that could be linked with neurodevelopmental milestone delays (and resulting ID): APP controls adult hippocampal neurogenesis, maintaining the tone of action of inhibitory GABAergic interneurons (Wang et al., 2014b). Interestingly, in the Ts65Dn partial trisomy 16 mice, defects in retrograde neurotrophin signaling and cholinergic forebrain neuronal degeneration are specifically related to extra APP gene dosage (Salehi et al., 2006). Similarly, endosomal abnormalities in the form of enlarged organelles which are characteristic changes in brains of both AD and DS individuals and also a consequence of increased APP expression (Cataldo et al., 2003). Whether these alterations are due to elevated APP or Aβ, or both, is not clear although there are suggestions from cultured neurons that endosomal dysfunction may be Aβ-independent (Jiang et al., 2010). Also, in lymphoblastoid cell lines carrying amyloid precursor protein (APP) microduplications causing autosomal dominant EOAD, enlarged endosomes were absent, suggesting that APP overexpression alone is not involved in the modification of early endosomes, but overexpression of other chromosome 21 genes plays an important role (Cossec et al., 2012). Finally, APP whole protein was also shown binding to Aβ, adding to the complexity of the potentially pathological interactions (Lorenzo et al., 2000).

## Why are vascular and mixed dementia not prevalent in DS?

Mixed dementia (MD)—defined as the coexistence of Alzheimer's disease (AD) and cerebrovascular disease (CVD) (Rockwood, 2003) has been identified as one of the most common subtypes of dementia by autopsy-based epidemiological studies (Skoog et al., 1993; Snowdon et al., 1997). The presence and degree of CVD modulates the cognitive picture of AD (Dong et al., 2013). Neuropathological studies have shown that infarcts increase the odds of dementia in patients with equivalent AD burden by adding to the deleterious effects of AD pathology (Schneider et al., 2007). While most common in hypertensive individuals, intracerebral hemorrhage has been reported in 20–50% of APP-Dup cases (Rovelet-Lecrux et al., 2006, 2007; Kasuga et al., 2009; Cohn-Hokke et al., 2012; McNaughton et al., 2012; Wallon et al., 2012), whereas individuals with DS are generally protected from this pathology. Reasons for this protection in DS individuals are unknown, but are potentially very important. Both dupAPP and DS show severe cerebral amyloid angiopathy (CAA) (Belza and Urich, 1986; McCarron et al., 1998; Rovelet-Lecrux et al., 2006, 2007; Kasuga et al., 2009; Cohn-Hokke et al., 2012; McNaughton et al., 2012; Wallon et al., 2012) which renders blood vessels more susceptible to vessel wall breakdown and subsequent hemorrhage. It remains to be answered whether more general disturbances of vascular physiology seen in DS (but not in dupAPP) have anything to do with the apparent protection from vascular dementia and higher frequency of cerebral hemorrhages. Several effects caused by T21 have been described that could affect vascular biology (Vis et al., 2009; Draheim et al., 2010). T21 has a powerful effect on inhibition of angiogenesis, and a reduced response to pro-angiogenic cytokines, such as VEGF-A (Arron et al., 2006). This biological feature of T21 is one of the explanations for the reduced incidence of solid tissue tumors in DS (Baek et al., 2009; Yang and Reeves, 2011; Nizetic and Groet, 2012). This is attributed to at least 7 genes on HSA21, and full mechanisms are not completely understood (Zorick et al., 2001; Ryeom et al., 2003; Arron et al., 2006; Baek et al., 2009; Reynolds et al., 2010; Yang and Reeves, 2011; Nizetic and Groet, 2012).

As regards atherosclerotic cardiovascular disease (Vis et al., 2009), although abnormalities in lipid metabolism, which are associated with high risk of premature atherosclerosis in the general population (increased triglycerides, decreased HDL), are frequently seen in patients with DS (Dörner et al., 1984; Bocconi et al., 1997; Corsi et al., 2005), and despite reduced physical activity and high rates of obesity (de Winter et al., 2012), atherosclerosis and coronary artery disease related mortality is surprisingly low (Baird and Sadovnick, 1988; Corsi et al., 2005; Lott and Head, 2005), a finding that led some authors to conclude that DS may represent an atheroma-free model of disease (Murdoch et al., 1977). Interestingly, hyper-triglyceridaemia, increased obesity and low exercise rates are common in adults with DS (Haas et al., 2013), and high cholesterol levels have been associated with risk of developing dementia (Chang et al., 2013). However, some cardiovascular risk factors, including hypertension, atherosclerosis, and smoking (McGowan et al., 2006; Troca-Marín et al., 2012) that are thought to contribute to the development of dementia in the general population (Schulz-Schaeffer, 2010), are lower among adults with DS.

Other DS-specific biological features may also play a role. While DS neurons show a severe down-regulation of mitochondrial function (Busciglio and Yankner, 1995; Roat et al., 2007; Murray et al., 2015), multiple studies indicate that reducing mitochondrial function can protect against aging and age-associated diseases (Trifunovic and Ventura, 2014). DS cells demonstrate adaptive down-regulation of mitochondrial function for survival under increased ROS conditions (Helguera et al., 2013).

## Is intensified neuronal activity good or bad for life-long DS cognitive functioning?

Recent work on mouse models has shown that hyperactivity of GABAergic interneurons in mouse models of DS over-inhibits hippocampal cortical excitatory neurons (Fernandez et al., 2007; Kleschevnikov et al., 2011). This has resulted in the first clinical trials in adults with DS for the improvement of cognitive functions, for the cognitive enhancement with GABA-α5 inverse agonists (Martínez-Cué et al., 2014). So, increased activity of one type of neurons is causing a decreased activity of another type of neurons in DS.

However, by increasing the neuronal activity of hippocampal cortical excitatory neurons, in theory, we also increase Aβ production and release. In fact, it has been demonstrated that both Aβ secretion, and synaptic plasticity are dependent on neuronal activity (Kamenetz et al., 2003; Cirrito et al., 2005; Scheff et al., 2006; Shrestha et al., 2006; Cheng et al., 2014; Lundgren et al., 2014). It remains unclear though whether inhibitory neurons have as much APP as excitatory neurons, or, whether over activity of inhibitory vs. excitatory neurons drive more Aβ generation. A proteolytic fragment (p25) of the cdk5-activator is generated as a function of neuronal activity, and it regulates synaptic plasticity and Aβ-induced cognitive impairment (Seo et al., 2014). Aβ-generation, endosomal trafficking and secretion (Wu et al., 2011; Sullivan et al., 2013; Lundgren et al., 2014) and Aβ-dependent Tau translocation to excitatory synapses (Frandemiche et al., 2014) are all neuronal activity-dependent. On the other hand, synaptic plasticity and synaptic maturation have also been shown to be neuronal activity-dependent processes (Fukazawa et al., 2003; Segal, 2005, 2010; Bosch and Hayashi, 2012; Heimer-McGinn et al., 2013; Ramiro-Cortés and Israely, 2013).

This seemingly controversial roles of neuronal activity level in DS pathology have also repercussions when it comes to therapeutic management approaches: sleep deprivation contributes to increased Aβ generation/secretion (Kang et al., 2009) as well as reduced clearance (Xie et al., 2013), and sleep deprivation in DS was shown to affect cognitive function (Brooks et al., 2015). On the other hand, deep brain stimulation was proposed as one of the intervention approaches to ameliorate cognitive dysfunction in AD (Boggio et al., 2011). More research is needed in this direction, as clearly opposing consequences could be reached, if the approaches are not better understood, and accordingly fine-tuned.

In conclusion, there are clearly many open questions on the inter-relation between pathogenic processes that affect neuronal development, synaptic plasticity, neuronal aging and longevity, and AD in DS. We have not had the space or scope in this mini-review to mention the potential modulating action of all other chromosome 21 genes that might influence this process, besides APP. Much more research is needed using high-resolution dissection of individual chr21 gene contributions using mouse models and human iPSC modeling. Such efforts should be coordinated and inter-disciplinary, including clinical dementia and cognitive assessments, and imaging studies.

### Conflict of interest statement

The authors declare that the research was conducted in the absence of any commercial or financial relationships that could be construed as a potential conflict of interest.
